# Data mining of adverse drug event signals with Nirmatrelvir/Ritonavir from FAERS

**DOI:** 10.1371/journal.pone.0316573

**Published:** 2024-12-31

**Authors:** Ji Sun, Xuanyu Deng, Juanjuan Huang, Gefei He, Shiqiong Huang

**Affiliations:** 1 The Affiliated Changsha Hospital of Xiangya School of Medicine, Central South University, Changsha, China; 2 The First Hospital of Changsha, Changsha, PR China; Peking University First Hospital, CHINA

## Abstract

Nirmatrelvir/Ritonavir, acting as an effective agent against COVID-19, has achieved considerable results in clinical studies in terms of drug efficacy. However, there is little research about its medication safety. Based on the FDA adverse event reporting system (FAERS) database, this study aims to mine the adverse reaction signals of the latest major recommended drug Nirmatrelvir/Ritonavir for the antiviral treatment of COVID-19, so as to provide a basis for safe and rational drug use. The reporting odds ratio (ROR) was used to explore the adverse event report data of all COVID-19 emergency use authorization (EUA) products in the FAERS database with the deadline of third quarter of 2023. In the analysis, 135427 adverse drug event (ADE) reports were found, and 35250 ADEs were reported with Nirmatrelvir/Ritonavir as the primary suspected drug, which was involved in multiple system. There was a high signal intensity of dysgeusia (ROR = 72.98), diarrhea (ROR = 3.03) and headache (ROR = 1.25), which was compatible with the adverse reactions recorded in the manual for Nirmatrelvir/Ritonavir. In addition, it was suggested that Nirmatrelvir/Ritonavir might cause pale-colored stools (ROR = 45.53), chromaturia (ROR = 3.07), yellow skin (ROR = 3.62), tongue coating (ROR = 35.55) and other new adverse reactions (not included in the instructions manual for Nirmatrelvir/Ritonavir). The ADEs of Nirmatrelvir/Ritonavir that are not in the instructions and are highly relevant in the real world are supplemented, prompting clinical attention to the ADEs of the drug, and providing a theoretical basis for the safe and effective application of the drug.

## Introduction

Nirmatrelvir/Ritonavir is a combination of two antiviral drugs, Nirmatrelvir and Ritonavir. Nirmatrelvir, mainly metabolized by CYP3A4 metabolic enzymes, can target the inhibition of 3CL protease to slow down the replication of the new coronavirus [1]. While Ritonavir can inhibit the decomposition and metabolism of Nirmatrelvir by blocking CYP3A4 enzymes, thereby improving its in vivo exposure level [1]. This synergy of the two drugs amplifies the antiviral effects.

Since its outbreak of COVID-19, COVID-19 has reached countries around the world, resulting in a large number of infections, severe illnesses and deaths [2, 3]. The disease, COVID-19, is characterized by a range of symptoms, primarily including dry throat, sore throat, cough and fever. Some patients may further suffer from muscle pain, loss of smell or taste, nasal congestion, runny nose, diarrhea, conjunctivitis [4]. In the long process of fighting COVID-19, the mutation of the virus never stopped, from the original strain of the virus that was first discovered, to the Omicron strain [5]. New corona-related clinical trials have been carried out around the world, and new drugs are constantly being developed and marketed [6]. As a new oral drug for COVID-19 [7–11], Nirmatrelvir/Ritonavir has achieved considerable results in clinical studies in terms of drug efficacy. This drug can reduce the risk of death or hospitalization of COVID-19 by 89% [12]. Nirmatrelvir/Ritonavir received the first emergency use grant in the United States on December 22, 2021 [13], which provided a new treatment scheme for mild to moderate COVID-19 patients with severe high-risk factors such as old age, chronic kidney disease, diabetes, cardiovascular disease, chronic lung disease, etc. However, as a new antiviral drug, the clinical record is not yet sufficient for its worldwide authorization, and there are even fewer reports on its adverse drug events (ADEs) [9, 14–16].

Spontaneous reporting is the most important adverse drug reaction monitoring method in the world at present. The database of adverse event spontaneous reporting system is an important data source for discovering adverse reaction signals [17, 18]. It can be helpful in not only discovering potential adverse reactions, but also finding new or rare adverse reactions. In this study, we detected and analyzed the signals of ADE reports after the authorized use of Nirmatrelvir/Ritonavir in FDA adverse event reporting system (FAERS), identified the potential high-risk safety signals, and provided reference basis for the clinical rational use of the drug in COVID-19 epidemic prevention and control.

## Methods

### Data source

FAERS is a publicly available database, which is composed of adverse event reports that were submitted to the FDA [9]. FAERS data contain demographic and administrative information (DEMO), drug information, coded adverse events (REAC), patient outcomes (OUTC), report sources (RPSR), therapy start and end dates for reported drugs (THER) and indications for drug administration (INDI). All the data can be accessed at https://fis.fda.gov/extensions/FPD-QDE-FAERS/FPD-QDE-FAERS.html. We downloaded the adverse event reports of all COVID-19 EUA products in the FAERS database as of Q3 2023, including Nirmatrelvir/Ritonavir, Remdesivir, Molnupiravir, Bebtelovimab, Tocilizumab, Sotrovimab, Bamlanivimab/Etesevimab, Baricitinib. We also obtained the list of drug combinations based on the file of drug information (S1 Table). In addition, we limited the study population, and only the indications that were COVID-19, COVID-19 treatment, SARS-CoV-2 test positive, coronavirus infection, and COVID-19 pneumonia, were included in the study. To ensure data integrity, the search terms were limited to the generic name of the target drug "Nirmatrelvir/Ritonavir" and the trade name "Paxlovid". The obtained data is imported into Microsoft office Excel 2019 to screen, collate, and reports including ADE signals such as drug label indications, operational or product management errors were excluded to reduce bias.

### Data standardization

In this study, the preferred term (PT) and system organ class (SOC) in Medical Dictionary for Regulatory Activities (MedDRA) terminology (version 26.0) were used to classify and describe the primary suspected ADR events. We firstly screened the signals of all PTs within “Nirmatrelvir/Ritonavir” (Standardized MedDRA Query). Besides, we gathered more information including use indication, age at the time of the event and concurrent medicine use in the reports that contained the ADEs considered possible signals for Nirmatrelvir/Ritonavir. Then, we removed signals unrelated to drugs such as various operational complications, product problems, social environment, and finally obtained the effective signal PTs and the corresponding SOC. The identified signals were also compared to the ADEs reported on the product labels on the FDA website. Each ADE reports gathered the following data: DEMO, drug information, REAC, OUTC, report year and reporter type.

### Data mining method

To determine the ADE signals of Nirmatrelvir/Ritonavir compared to other drugs in the FAERS, we performed disproportionality analysis, so-called case/non-case analysis, which was a usually used method for signal detection in pharmacovigilance [19]. Two different disproportionate measures, namely, the ROR and information component (IC), were used to decrease the possibility of false-positive results. The formulas of ROR and IC was shown in S2 Table. Significant disproportionality was identified as the lower limit of the 95% confidence interval of ROR (ROR_025_) > 1 or the lower limit of the 95% confidence interval of IC (IC_025_) > 0, with reported cases > 3. In order to improve the reliability of our findings and minimize these potential biases, we employed the multivariable logistic regression to adjust ROR. The top three drugs in the list of drug combinations were included into the regression model. Statistical analyses were carried out using IBM® SPSS® Statistics software (version 27.0) and R (version 4.3).

### Clinical prioritization evaluation

A semiquantitative assessment of emerging signals within the PT was conducted to evaluate their clinical prioritization across five dimensions: number of reports, lower limit of ROR, mortality proportion, adherence to important medical events (IMEs) or designated medical events (DMEs) criteria, and biological plausibility (S3 Table) [20, 21]. IMEs and DMEs were established and standardized by the European Medicines Agency [22, 23]. AEs with cumulative scores of 0–4, 5–7, or 8–10 were designated as weak, moderate, or strong clinical priorities, respectively.

## Results

### Basic information of ADE reports

A total of 135427 adverse event reports for all COVID-19 EUA products were retrieved and screened. Among them, there were 35250 ADE reports in which Nirmatrelvir/Ritonavir was suspected as the primary drug. The basic information regarding gender, age, country of occurrence of ADE and reporter is shown in Table 1.

**Table 1:** Basic information of adverse drug event reports about Nirmatrelvir/Ritonavir.

Basic information	Classification	ADE cases	Proportion (%)
Gender	Male	11432	32.43
	Female	20450	58.01
	Not specified	3368	9.55
Age	< 18 years	298	0.85
	18–64 years	15938	45.21
	≥ 65 years	12269	34.81
	Not specified	6745	19.13
Country where event occurred	America	32294	91.61
	Europe	1667	4.73
	Asia	1163	3.30
	Africa	11	0.03
	Oceania	114	0.32
	Not specified	1	0.00
Reporter Type	Consumer	1733	4.92
	Healthcare professional	766	2.17
	Not specified	32751	92.91
Total		35250	

The basic information of ADE reports for Nirmatrelvir/Ritonavir are presented in Table 1, showcasing their characteristics. Among the reports, the largest proportion (45.21%) were those aged 18–64 years. Females (58.01%) accounted for a higher proportion compared to males (32.43%), and 4.92% of the reports were provided by consumers, 2.17% by healthcare professionals, and the remaining reports (92.91%) did not provide information about the reporter. In terms of geography, America had the largest percentage of reports (91.61%), with Europe (4.73%), Asia (3.30%), Oceania (0.32%), and Africa (0.03%) following closely behind. The numbers and proportions of PT for Nirmatrelvir/Ritonavir and comparator drugs were shown in S4 Table. Besides, the distribution of common ADE reports for all other COVID-19 EUA products for comparison to Nirmatrelvir/Ritonavir was shown in S1 Fig.

### ADE signals involving system organ classes

**Fig 1:**
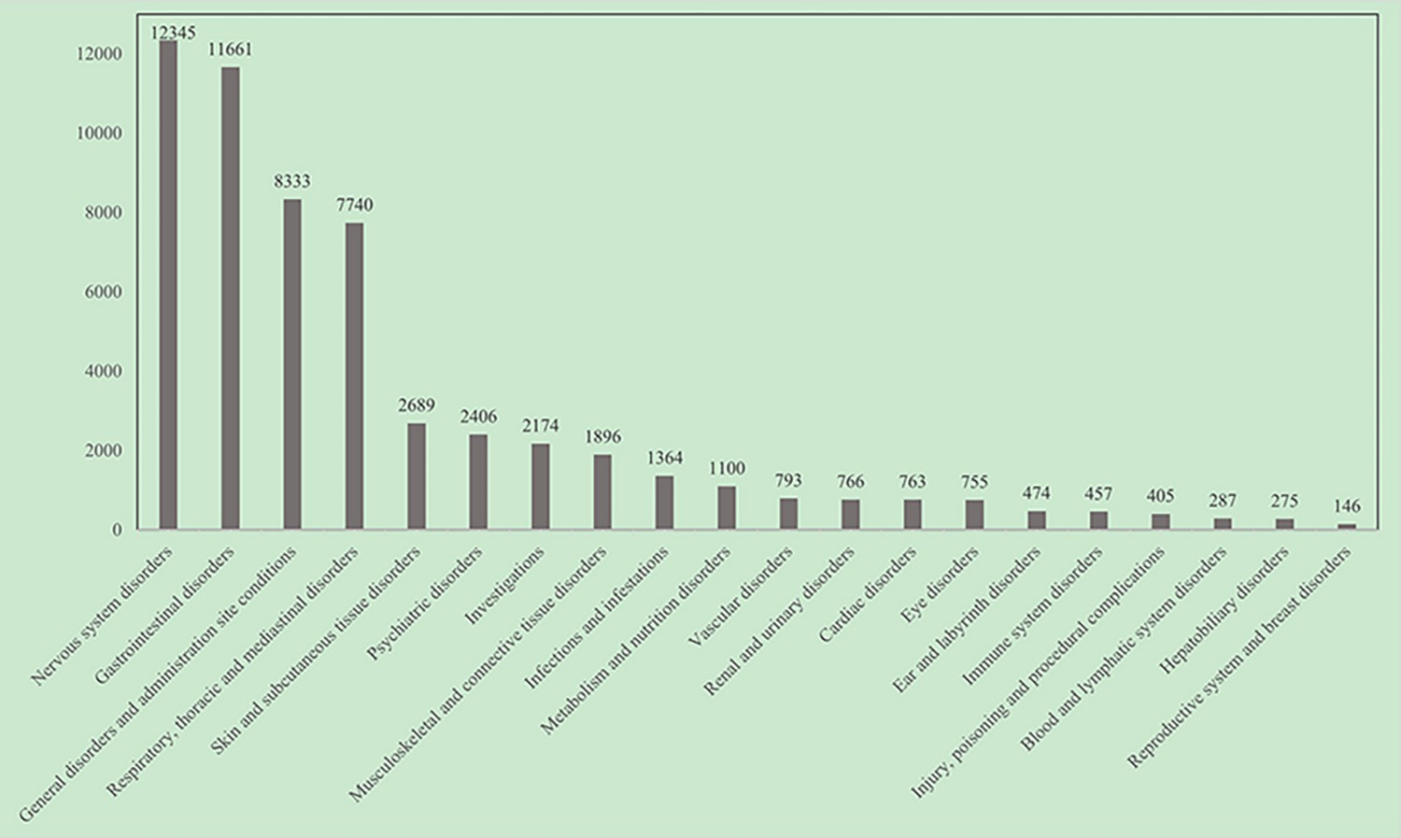
Classification of system organs involved in the adverse drug event signal of Nirmatrelvir/Ritonavir.

A total of 35250 Nirmatrelvir/Ritonavir ADE signals were screened from 57090 ADE reports involving 26 SOCs. As shown in Fig 1, we listed the top 20 SOCs. The cumulative number of ADE reports of "nervous system disorders" in SOC was as high as 12345 cases (21.62%), followed by the gastrointestinal disorders (20.43%), and general disorders and administration site conditions (14.60%).

### Primary analysis

To mitigate the influence of potential confounders, such as medication combinations, we incorporated the top 3 combined drugs (Levothyroxine, Atorvastatin and Lisinopril) as confounding variables, along with the target drug Nirmatrelvir/Ritonavir, in a multivariable logistic regression model.

**Fig 2:**
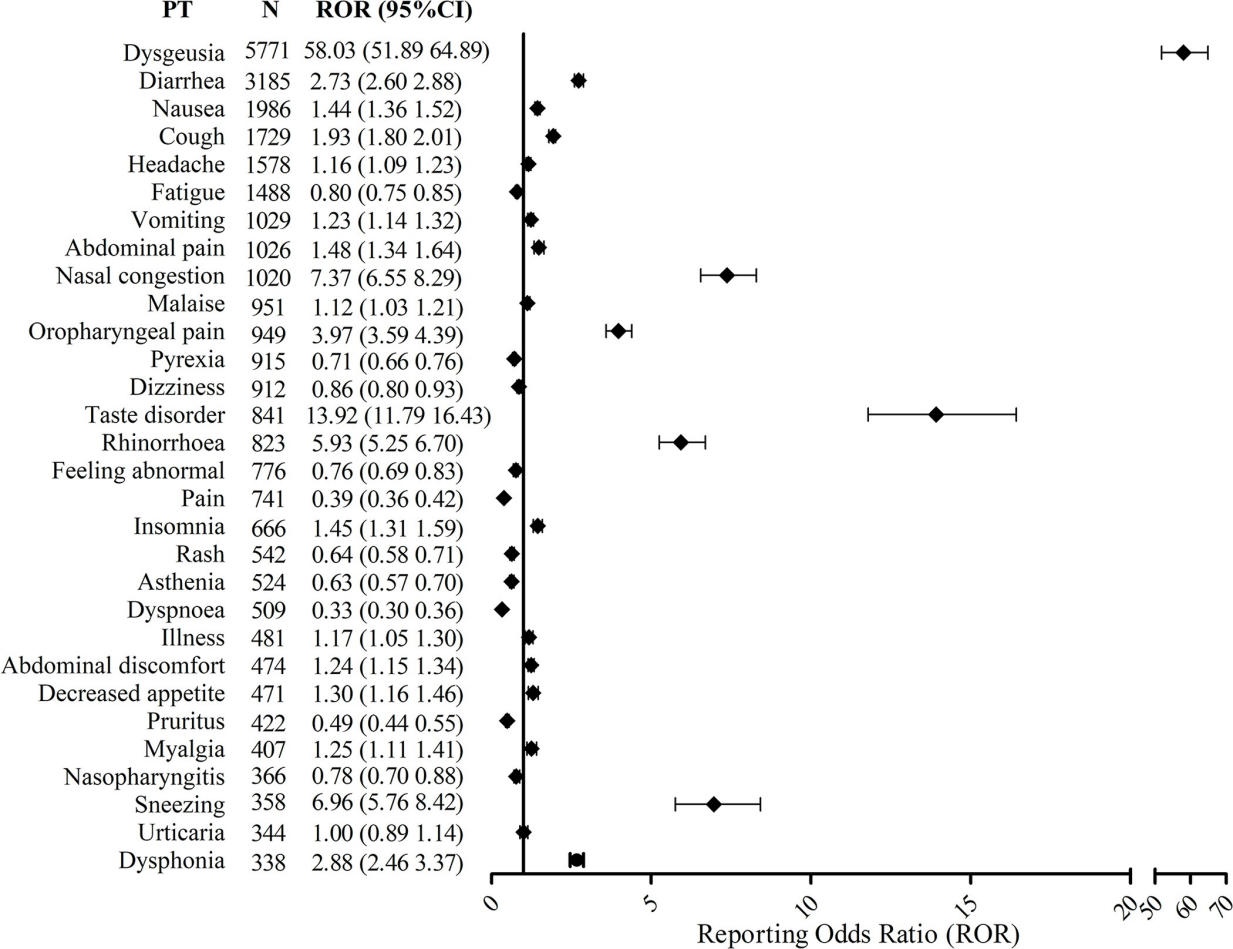
Signals of Nirmatrelvir/Ritonavir based on the number of preferred terms in adverse event reporting system. PT, preferred term. N, number of preferred terms. ROR, reporting odds ratio.

ADE signals of Nirmatrelvir/Ritonavir based on the number of PTs were shown in Fig 2. Among top 30 PTs, there was a high signal intensity of dysgeusia (ROR: 72.98, adjusted ROR: 58.03), diarrhea (ROR: 3.03, adjusted ROR: 2.73), nausea (ROR: 1.57, adjusted ROR: 1.44), headache (ROR: 1.25, adjusted ROR: 1.16), vomiting (ROR: 1.39, adjusted ROR: 1.2), abdominal pain (ROR: 1.27, adjusted ROR: 1.48) and malaise (ROR: 1.21, adjusted ROR: 1.12), which was compatible with the adverse reactions recorded in the manual for Nirmatrelvir/Ritonavir (Fig 2). Furthermore, it was shown that Nirmatrelvir/Ritonavir might cause cough (ROR: 3.37, adjusted ROR: 1.93) and nasal congestion (ROR: 8.60, adjusted ROR: 7.37), which was not included in the instruction’s manual for Nirmatrelvir/Ritonavir.

**Fig 3:**
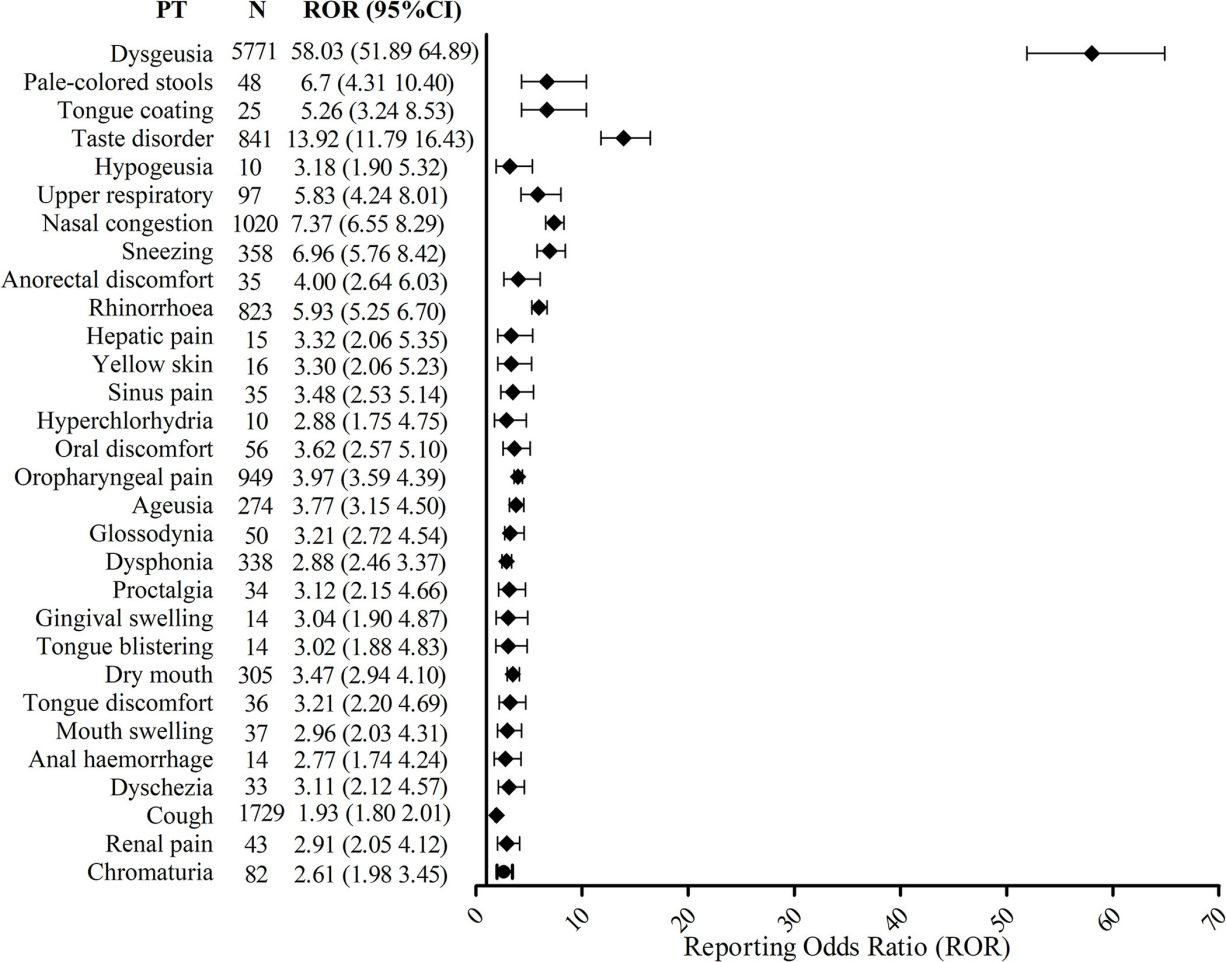
Signals of Nirmatrelvir/Ritonavir based on the signal intensity of preferred terms in FDA adverse event reporting system. PT, preferred term. N, number of preferred terms. ROR, reporting odds ratio.

In addition, the ADE signals of Nirmatrelvir/Ritonavir based on the ROR level were shown in Fig 3. Dysgeusia also ranked the first in the signal intensity of ADE (ROR: 72.89, adjusted ROR: 58.03). In addition, as the adverse reactions mentioned in the instruction manual, pale-colored stools (ROR: 45.53, adjusted ROR: 6.7) and tongue coating (ROR: 35.55, adjusted ROR: 5.26), had significant signal strength. However, other adverse reactions with high signal intensity, such as taste disorder (ROR: 22.23, adjusted ROR: 13.92), hypogeusia (ROR: 9.48, adjusted ROR: 3.18), yellow skin (ROR: 5.05, adjusted ROR: 3.30), chromaturia (ROR: 3.07, adjusted ROR: 2.61) and gingival swelling (ROR: 3.98, adjusted ROR: 3.04), were not mentioned in the instructions.

### Clinical prioritization analysis of signals

In the above results, the 30 PTs were selected for clinical priority assessment (Table 2). Three of them were categorized as IMEs, including dysgeusia, taste disorder and ageusia. Dysgeusia, pale-colored stools, dry mouth, yellow skin, mouth swelling and chromaturia were evaluated as strong clinical evidence of “++”. Based on the clinical priority assessment, dysgeusia was rated as moderate clinical priority, with a maximum priority of 7 points.

**Table 2:** Clinical priority assessing results of disproportionality signals.

PTs	N	Adjusted ROR	Death (n)	IMEs or DMEs	Relevant evidence evaluation	Priority level (score)
Dysgeusia	5771	51.89	2	IME	++	Moderate (7)
Taste disorder	841	11.79	1	IME	-	Moderate (5)
Nasal congestion	1020	6.55	0	None	-	Weak (4)
Sneezing	358	5.76	0	None	-	Weak (4)
Rhinorrhoea	823	5.25	5	None	-	Weak (4)
Pale-colored stools	48	4.31	0	None	++	Weak (4)
Upper respiratory tract congestion	97	4.24	0	None	-	Weak (3)
Oropharyngeal pain	949	3.59	9	None	-	Weak (3)
Tongue coating	25	3.24	0	None	-	Weak (2)
Ageusia	274	3.15	4	IME	-	Weak (4)
Dry mouth	305	2.94	0	None	++	Moderate (5)
Glossodynia	50	2.72	0	None	-	Weak (2)
Anorectal discomfort	35	2.64	0	None	-	Weak (2)
Oral discomfort	56	2.57	0	None	-	Weak (3)
Dysphonia	338	2.46	2	None	-	Weak (3)
Sinus pain	35	2.35	0	None	-	Weak (2)
Tongue discomfort	36	2.20	0	None	-	Weak (2)
Proctalgia	34	2.15	0	None	-	Weak (2)
Dyschezia	33	2.12	0	None	-	Weak (2)
Hepatic pain	15	2.06	0	None	-	Weak (2)
Yellow skin	16	2.06	0	None	++	Weak (4)
Renal pain	43	2.05	0	None	-	Weak (2)
Mouth swelling	37	2.03	0	None	++	Weak (4)
Chromaturia	82	1.98	1	None	++	Weak (4)
Hypogeusia	10	1.9	0	None	-	Weak (0)
Gingival swelling	14	1.90	0	None	-	Weak (0)
Tongue blistering	14	1.88	0	None	-	Weak (0)
Cough	1729	1.8	0	None	-	Weak (1)
Hyperchlorhydria	10	1.75	0	None	-	Weak (0)
Anal haemorrhage	14	1.74	0	None	-	Weak (0)

Mortality proportion: percentage of cases in which death was reported as an outcome in the overall cases report for a particular adverse event. IMEs and DMEs are developed and updated by EMA (European Medicines Agency). ++: AEs are mainly from the FDA Prescribing Information, Phase 2/3 RCTs, or systematic reviews, with biological plausibility. +: AEs are mainly from other clinical trials, observational studies, or case reports/series with potential biological plausibility. -: AEs only emerging from disproportionality analyses. PT, preferred term. N, number of reports. AEs, adverse events. DMEs, designated medical events. IMEs, important medical events. MHRA, medicine and healthcare products regulatory agency. RCTs, randomized controlled trials.

## Discussion

After the comparison of the screened effective ADR signals with the instructions of Nirmatrelvir/Ritonavir, several novel ADRs were found. There are 20 of the top 30 ADR signals in terms of PTs that are not mentioned in the instruction manual for Nirmatrelvir/Ritonavir, and 27 of the top 30 ADR signals in terms of ROR are not mentioned in the instructions. These results provide a reference basis for further updating the ADR in the instructions of Nirmatrelvir/Ritonavir. We also discovered that the detected ADE signal of Nirmatrelvir/Ritonavir was consistent with the drug instructions and result in another study [15, 24]. The common adverse reactions mentioned in the instructions of Nirmatrelvir/Ritonavir are diarrhea and dysgeusia. Among 35250 ADE reports in our study, there were 5771 dysgeusia and 3185 diarrheas, accounting for 16.37% and 9.04% respectively, which was compatible with the instructions. Most of adverse reactions mentioned in the instructions of Nirmatrelvir/Ritonavir and clinical trials [15, 24] were confirmed in this study, which further verified the reliability of this study. In addition, the study also found a number of ADEs that were not mentioned in the manual for Nirmatrelvir/Ritonavir, such as taste disorder, nasal congestion and other liver and kidney dysfunction, which should be paid more attention in the future.

In the Fig 2, we found that the number of ADE reports in SOC with other special sensory impairment was relatively high, especially the dysgeusia signal. Our study found that 5771 of the 35250 ADE reports had dysgeusia, accounting for 16.37%. This is consistent with the common adverse reactions mentioned in the drug description. However, adverse reactions not mentioned in the instructions, such as ageusia, taste disorder, and hypergeusia, were strong ADE signals of Nirmatrelvir/Ritonavir. At present, the mechanisms were not fully clarified in terms of Nirmatrelvir/Ritonavir causing such ADRs. The possible mechanism was that the structure of the drug itself impaired the sense of taste. Notably, neuropathy was one of the common features of COVID-19, suggesting that the olfactory or taste nerves were neuroinvasive [25–27]. Therefore, when a COVID-19 patient had gustatory problem, the clinician should determine whether it was due to the drug’s adverse reaction or the patient’s disease. When Nirmatrelvir/Ritonavir was used in patients who had experienced taste problems, care should be taken in case because it might worsen the patient’s taste problems.

It was worth noting that the signal of tongue coating, glossodynia, oral discomfort, tongue blistering, tongue discomfort, and gingival swelling were strong ADE signals of Nirmatrelvir/Ritonavir, but this was also not mentioned in the instructions. ACE2, a host cell receptor expressed in lung cells and multiple extra pulmonary tissues [28, 29], was highly expressed in oral mucosa, which was critical for SARS-CoV-2 infection [30]. Although few ADEs associated with Nirmatrelvir/Ritonavir had been reported, they showed a strong association with the drug in our study. Future studies are needed to attract more attention to the ADE of gums and oral cavity caused by the Nirmatrelvir/Ritonavir. Although the effects of Nirmatrelvir/Ritonavir on liver and kidney function are not mentioned in the instructions, previous studies have also shown minimal effects on liver and kidney function [31]. In our study, signs and symptoms of liver problems such as pale-colored stools, hepatic pain, and yellow skin (jaundice) were found to signal adverse reactions. These reported ADE signals were small in number, but high in intensity, such as pale-colored stools (ROR: 45.53), hepatic pain (ROR: 5.33), and yellow skin (ROR: 5.05), suggesting that it was easy to ignore these ADE signals. Therefore, healthcare workers should pay more attention to patient’s liver-related symptoms and signs when prescribing Nirmatrelvir/Ritonavir.

Additionally, there were also strong ADE signals of urinary system dysfunction such as chromaturia and renal pain, but the dysfunction of the drug to the urinary system was not mentioned in the specification. Although there is currently no plausible explanation for the mechanism of these new ADE signals, a related mechanism of Nirmatrelvir/Ritonavir drug interaction may provide a partial explanation. Since Nirmatrelvir was mainly excreted through the kidney in prototype [1], the dose should be adjusted strictly according to the level of renal function in clinical use. When the drug used in patients with moderate renal insufficiency, the level of renal function should be closely monitored to prevent dysfunction and protect urinary system from potential drug-related dysfunction.

We also performed a clinical prioritization assessment to prioritize the ADE signals. Among the 30 confirmed PT signals, none were classified as high clinical priority. Although dysgeusia, taste disorder and dry mouth were rated as moderate clinical priority, investigation into their clinical prioritization disclosed their prevalence and previous disclosure or reporting.

Through the analysis of vast data of FAERS, this study can obtain the medication data in the real world and effectively solve the problems of small sample size and short observation time of clinical trials. However, there are still some limitations. FAERS database is a spontaneous reporting system of ADE [13], some drawbacks are inevitable such as randomness, underreporting, and inaccurate reporting, which may lead to deviation of the research results. Moreover, due to the undetailed information of patients with medication, the incidence of ADE of Nirmatrelvir/Ritonavir cannot be calculated. Finally, the existence of reports on specific drugs or biological products in the database cannot determine whether the ADE is caused by certain drug. For any given ADE report, the ADE signal calculated by the proportional imbalance method indicates that the target drug is statistically related to the target ADE, but not biologically related, which does not prove that the target drug has a definite causal relationship with the target ADE. Therefore, further meticulous investigations are imperative through the conduits of clinical trials.

## Conclusion

The present study not only identified ADEs similar to those in the instruction of Nirmatrelvir/Ritonavir, but also explored certain new ADEs, such as pale-colored stools, gingival swelling, yellow skin and chromaturia, which contributed to prompting clinical attention to the ADEs of Nirmatrelvir/Ritonavir and providing a theoretical basis for the safe and effective application of the drug.

## Supporting information

S1 Fig The distribution of common adverse drug events reported for all other COVID-19 EUA products for comparison to Nirmatrelvir/Ritonavir. PT, preferred term. N, number of preferred terms.
(TIF)

S1 Table The list of top l0 concomitant drugs. (DOCX)

S2 Table The formulas of reporting odds ratio and information component. (DOCX)

S3 Table A rating scale assessing clinical priority of disproportionality signals. (DOC)

S4 Table The numbers and proportions of preferred term for Nirmatrelvir/Ritonavir and comparator drugs. (DOCX)
