# Supplementary figures and images for: Data mining of adverse drug event signals with Nirmatrelvir/Ritonavir from FAERS
Source: PLoS One. 2024 Dec 31;19(12):e0316573. doi: 10.1371/journal.pone.0316573 (PMC11687713; doi:10.1371/journal.pone.0316573)

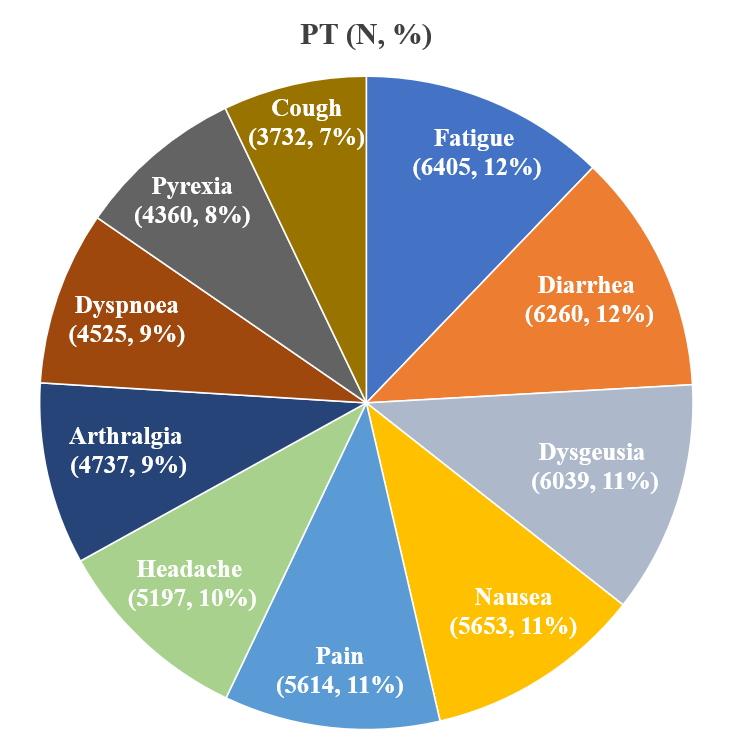

Supplement: S1 Fig — PT, preferred term. N, number of preferred terms. (TIF) [file pone.0316573.s001.tif]
