# Supplementary material for: Data mining of adverse drug event signals with Nirmatrelvir/Ritonavir from FAERS
Source: PLoS One. 2024 Dec 31;19(12):e0316573. doi: 10.1371/journal.pone.0316573 (PMC11687713; doi:10.1371/journal.pone.0316573)
Supplement: S1 Table — (DOCX) [file pone.0316573.s002.docx]

| **Concomitant drugs** | **Number** | **Proportion (%)** |
| --- | --- | --- |
| Levothyroxine | 1101 | 3.12 |
| Atorvastatin | 1087 | 3.08 |
| Lisinopril | 912 | 2.59 |
| Aspirin | 824 | 2.34 |
| Losartan | 778 | 2.21 |
| Tylenol | 689 | 1.95 |
| Omeprazole | 664 | 1.88 |
| Amlodipine | 643 | 1.82 |
| Metformin | 639 | 1.81 |
| Vitamin | 598 | 1.70 |

**S1 Table. The list of top l0 concomitant drugs.**
