# Supplementary material for: Data mining of adverse drug event signals with Nirmatrelvir/Ritonavir from FAERS
Source: PLoS One. 2024 Dec 31;19(12):e0316573. doi: 10.1371/journal.pone.0316573 (PMC11687713; doi:10.1371/journal.pone.0316573)
Supplement: S2 Table — (DOCX) [file pone.0316573.s003.docx]

**S2 Table. The formulas of reporting odds ratio and information component.**

| **Algorithms** | **Equation** | **Criteria** |
| --- | --- | --- |
| ROR | ROR=ad/bc | ROR_025_ > 1 |
|  | 95%CI=e^ln(ROR)±1.96(1/a+1/b+1/c+1/d)∧0.5^ |  |
| IC | IC=log_2_a(a+b+c+d)(a+c)(a+b) | IC_025_ > 0 |
|  | 95%CI= E(IC)±2V(IC)^0.5 |  |

Abbreviations: a, number of reports containing both the target drug and target adverse event; b, number of reports containing other adverse event of the target drug; c, number of reports containing the target adverse event of other drugs; d, number of reports containing other drugs and other adverse event; 95%C1, 95% confidence interval.
