# Supplementary material for: Data mining of adverse drug event signals with Nirmatrelvir/Ritonavir from FAERS
Source: PLoS One. 2024 Dec 31;19(12):e0316573. doi: 10.1371/journal.pone.0316573 (PMC11687713; doi:10.1371/journal.pone.0316573)
Supplement: S3 Table — (DOC) [file pone.0316573.s004.doc]

**S3 Table. A rating scale assessing clinical priority of disproportionality signals.**

| **Assessment items** | **2 points** | **1 point** | **0 point** |
| --- | --- | --- | --- |
| Number of target events | >50 | 10-50 | <10 |
| Lower limit of ROR | >5 | 2-5 | 1-2 |
| Mortality proportion (%) | >50 | 25-50 | <25 |
| IMEs or DMEs | DME | IME | None |
| Current evidence evaluation | ++ | + | **-** |

Mortality proportion: percentage of cases in which death was reported as an outcome in the overall cases report for a particular adverse event. IMEs and DMEs are developed and updated by EMA (European Medicines Agency). ++: AEs are mainly from the FDA Prescribing Information, Phase 2/3 RCTs, or systematic reviews, with biological plausibility. +: AEs are mainly from other clinical trials, observational studies, or case reports/series with potential biological plausibility. -: AEs only emerging from disproportionality analyses. AEs, adverse events. DMEs, designated medical events. IMEs, important medical events. MHRA, medicine and healthcare products regulatory agency. RCTs, randomized controlled trials.
