# Supplementary material for: Data mining of adverse drug event signals with Nirmatrelvir/Ritonavir from FAERS
Source: PLoS One. 2024 Dec 31;19(12):e0316573. doi: 10.1371/journal.pone.0316573 (PMC11687713; doi:10.1371/journal.pone.0316573)
Supplement: S4 Table — (DOCX) [file pone.0316573.s005.docx]

**S4 Table.** **The numbers and proportions of preferred term for Nirmatrelvir/Ritonavir and** **comparator drugs.**

| **PT** | **Nirmatrelvir/Ritonavir and Comparator drugs** | **Number of target ADE reports** | **Proportion (%)** |
| --- | --- | --- | --- |
| Dysgeusia | Nirmatrelvir/Ritonavir | 5771 | 16.37 |
|  | Comparator drugs | 268 | 0.27 |
| Diarrhea | Nirmatrelvir/Ritonavir | 3185 | 9.04 |
|  | Comparator drugs | 3177 | 3.17 |
| Nausea | Nirmatrelvir/Ritonavir | 1986 | 5.63 |
|  | Comparator drugs | 3667 | 3.66 |
| Cough | Nirmatrelvir/Ritonavir | 1729 | 4.90 |
|  | Comparator drugs | 1508 | 1.51 |
| Headache | Nirmatrelvir/Ritonavir | 1578 | 4.48 |
|  | Comparator drugs | 3619 | 3.61 |
| Fatigue | Nirmatrelvir/Ritonavir | 1488 | 4.22 |
|  | Comparator drugs | 4917 | 4.91 |
| Vomiting | Nirmatrelvir/Ritonavir | 1029 | 2.92 |
|  | Comparator drugs | 2130 | 2.13 |
| Abdominal pain | Nirmatrelvir/Ritonavir | 1026 | 2.91 |
|  | Comparator drugs | 2310 | 2.31 |
| Nasal congestion | Nirmatrelvir/Ritonavir | 1020 | 2.89 |
|  | Comparator drugs | 346 | 0.35 |
| Malaise | Nirmatrelvir/Ritonavir | 951 | 2.70 |
|  | Comparator drugs | 2246 | 2.24 |
| Oropharyngeal pain | Nirmatrelvir/Ritonavir | 949 | 2.69 |
|  | Comparator drugs | 621 | 0.62 |
| Pyrexia | Nirmatrelvir/Ritonavir | 915 | 2.60 |
|  | Comparator drugs | 3445 | 3.44 |
| Dizziness | Nirmatrelvir/Ritonavir | 912 | 2.59 |
|  | Comparator drugs | 2731 | 2.73 |
| Taste disorder | Nirmatrelvir/Ritonavir | 841 | 2.39 |
|  | Comparator drugs | 110 | 0.11 |
| Rhinorrhea | Nirmatrelvir/Ritonavir | 823 | 2.33 |
|  | Comparator drugs | 344 | 0.34 |
| Feeling abnormal | Nirmatrelvir/Ritonavir | 776 | 2.20 |
|  | Comparator drugs | 1503 | 1.50 |
| Pain | Nirmatrelvir/Ritonavir | 741 | 2.10 |
|  | Comparator drugs | 4873 | 4.86 |
| Insomnia | Nirmatrelvir/Ritonavir | 666 | 1.89 |
|  | Comparator drugs | 1168 | 1.17 |
| Rash | Nirmatrelvir/Ritonavir | 542 | 1.54 |
|  | Comparator drugs | 2231 | 2.23 |
| Asthenia | Nirmatrelvir/Ritonavir | 524 | 1.49 |
|  | Comparator drugs | 1965 | 1.96 |
| Dyspnoea | Nirmatrelvir/Ritonavir | 509 | 1.44 |
|  | Comparator drugs | 4016 | 4.01 |
| Illness | Nirmatrelvir/Ritonavir | 481 | 1.36 |
|  | Comparator drugs | 1103 | 1.10 |
| Abdominal discomfort | Nirmatrelvir/Ritonavir | 474 | 1.34 |
|  | Comparator drugs | 947 | 0.95 |
| Decreased appetite | Nirmatrelvir/Ritonavir | 471 | 1.34 |
|  | Comparator drugs | 894 | 0.89 |
| Pruritus | Nirmatrelvir/Ritonavir | 422 | 1.20 |
|  | Comparator drugs | 2261 | 2.26 |
| Myalgia | Nirmatrelvir/Ritonavir | 407 | 1.15 |
|  | Comparator drugs | 874 | 0.87 |
| Nasopharyngitis | Nirmatrelvir/Ritonavir | 366 | 1.04 |
|  | Comparator drugs | 1292 | 1.29 |
| Sneezing | Nirmatrelvir/Ritonavir | 358 | 1.02 |
|  | Comparator drugs | 123 | 0.12 |
| Urticaria | Nirmatrelvir/Ritonavir | 344 | 0.98 |
|  | Comparator drugs | 946 | 0.94 |
| Dysphonia | Nirmatrelvir/Ritonavir | 338 | 0.96 |
|  | Comparator drugs | 235 | 0.23 |
| Pale-colored stools | Nirmatrelvir/Ritonavir | 48 | 0.14 |
|  | Comparator drugs | 3 | 0.003 |
| Tongue coating | Nirmatrelvir/Ritonavir | 25 | 0.07 |
|  | Comparator drugs | 2 | 0.002 |
| Hypogeusia | Nirmatrelvir/Ritonavir | 10 | 0.03 |
|  | Comparator drugs | 3 | 0.003 |
| Upper respiratory tract congestion | Nirmatrelvir/Ritonavir | 97 | 0.28 |
|  | Comparator drugs | 32 | 0.03 |
| Anorectal discomfort | Nirmatrelvir/Ritonavir | 35 | 0.10 |
|  | Comparator drugs | 14 | 0.01 |
| Hepatic pain | Nirmatrelvir/Ritonavir | 15 | 0.04 |
|  | Comparator drugs | 8 | 0.01 |
| Yellow skin | Nirmatrelvir/Ritonavir | 16 | 0.05 |
|  | Comparator drugs | 9 | 0.01 |
| Sinus pain | Nirmatrelvir/Ritonavir | 35 | 0.10 |
|  | Comparator drugs | 21 | 0.02 |
| Hyperchlorhydria | Nirmatrelvir/Ritonavir | 10 | 0.03 |
|  | Comparator drugs | 6 | 0.01 |
| Oral discomfort | Nirmatrelvir/Ritonavir | 56 | 0.16 |
|  | Comparator drugs | 34 | 0.03 |
| Ageusia | Nirmatrelvir/Ritonavir | 274 | 0.78 |
|  | Comparator drugs | 184 | 0.18 |
| Glossodynia | Nirmatrelvir/Ritonavir | 50 | 0.14 |
|  | Comparator drugs | 34 | 0.03 |
| Proctalgia | Nirmatrelvir/Ritonavir | 34 | 0.10 |
|  | Comparator drugs | 24 | 0.02 |
| Tongue blistering | Nirmatrelvir/Ritonavir | 14 | 0.04 |
|  | Comparator drugs | 10 | 0.01 |
| Gingival swelling | Nirmatrelvir/Ritonavir | 14 | 0.04 |
|  | Comparator drugs | 10 | 0.01 |
| Dry mouth | Nirmatrelvir/Ritonavir | 305 | 0.87 |
|  | Comparator drugs | 221 | 0.22 |
| Tongue discomfort | Nirmatrelvir/Ritonavir | 36 | 0.10 |
|  | Comparator drugs | 26 | 0.03 |
| Mouth swelling | Nirmatrelvir/Ritonavir | 37 | 0.10 |
|  | Comparator drugs | 29 | 0.03 |
| Anal haemorrhage | Nirmatrelvir/Ritonavir | 14 | 0.04 |
|  | Comparator drugs | 11 | 0.01 |
| Dyschezia | Nirmatrelvir/Ritonavir | 33 | 0.09 |
|  | Comparator drugs | 27 | 0.03 |
| Renal pain | Nirmatrelvir/Ritonavir | 43 | 0.12 |
|  | Comparator drugs | 38 | 0.04 |
| Chromaturia | Nirmatrelvir/Ritonavir | 82 | 0.23 |
|  | Comparator drugs | 76 | 0.08 |
